# Supplementary material for: A Likelihood Approach for Real-Time Calibration of Stochastic Compartmental Epidemic Models
Source: PLoS Comput Biol. 2017 Jan 17;13(1):e1005257. doi: 10.1371/journal.pcbi.1005257 (PMC5240920; doi:10.1371/journal.pcbi.1005257)
Supplement: S1 File — (TAR.GZ) [file pcbi.1005257.s014.tar.gz › HSPH_Online-SI-Revision/IPoi09/n1-severe/n1-severe_BENCH2_table8.pdf]

1	newly 3 weeks spec	10.	14.6207	{3., 26.}	82.069	0.278703	newly 3 weeks cumul	63.	53.0345	{20., 106.}	47.3454	0.249314	newly 5 weeks spec	19.	8.68966	{2., 20.}	66.4247	0.543448	newly 5 weeks cumul	93.	73.431	{28., 145.}	50.1854	0.270578	newly 46 weeks cumul	179.	111.741	{34., 200.}	58.0331	0.368923
2	newly 3 weeks spec	13.	10.8136	{2., 24.}	70.2738	0.409046	newly 3 weeks cumul	51.	42.339	{12., 85.}	61.5819	0.333221	newly 5 weeks spec	4.	7.69492	{1., 15.}	132.203	0.402838	newly 5 weeks cumul	64.	60.4576	{17., 116.}	64.7511	0.313093	newly 48 weeks cumul	85.	89.2542	{24., 168.}	72.0837	0.317085
3	newly 3 weeks spec	16.	20.6949	{8., 34.}	57.9449	0.210595	newly 3 weeks cumul	56.	75.1356	{31., 147.}	53.6017	0.183722	newly 5 weeks spec	18.	14.4746	{4., 26.}	54.2373	0.317396	newly 5 weeks cumul	91.	107.017	{41., 210.}	45.3157	0.171979	newly 47 weeks cumul	131.	166.017	{67., 303.}	53.4869	0.181271
4	newly 3 weeks spec	8.	17.2154	{6., 28.}	125.577	0.303107	newly 3 weeks cumul	27.	66.2769	{34., 110.}	145.698	0.326259	newly 5 weeks spec	8.	9.92308	{3., 15.}	65.9615	0.244503	newly 5 weeks cumul	40.	90.8462	{44., 140.}	128.731	0.291431	newly 50 weeks cumul	56.	124.708	{54., 184.}	125.824	0.282235
5	newly 3 weeks spec	13.	13.3733	{5., 21.}	40.	0.190266	newly 3 weeks cumul	49.	51.2933	{25., 77.}	35.483	0.153419	newly 5 weeks spec	5.	8.2	{3., 13.}	81.0667	0.248066	newly 5 weeks cumul	61.	69.6133	{35., 111.}	39.7814	0.156043	newly 49 weeks cumul	65.	93.6667	{48., 158.}	56.5744	0.179748
6	newly 3 weeks spec	15.	17.2222	{5., 33.}	51.0053	0.215054	newly 3 weeks cumul	74.	64.6984	{28., 111.}	37.752	0.200523	newly 5 weeks spec	23.	12.6984	{5., 23.}	57.902	0.414966	newly 5 weeks cumul	114.	91.619	{42., 160.}	41.242	0.226538	newly 46 weeks cumul	183.	155.698	{62., 260.}	47.6017	0.240736
7	newly 3 weeks spec	8.	8.92647	{3., 15.}	55.6985	0.245208	newly 3 weeks cumul	25.	34.6029	{14., 59.}	63.7059	0.209583	newly 5 weeks spec	2.	5.67647	{1., 11.}	207.353	0.467967	newly 5 weeks cumul	27.	47.8676	{17., 76.}	92.1024	0.248944	newly 46 weeks cumul	44.	71.2353	{26., 124.}	84.4251	0.246488
8	newly 3 weeks spec	0.1	8.13514	{3., 15.}	8035.14	1.82116	newly 3 weeks cumul	0.1	35.3784	{17., 63.}	35278.4	2.49441	newly 5 weeks spec	0.1	4.7973	{1., 11.}	4702.7	1.52119	newly 5 weeks cumul	0.1	47.2838	{24., 87.}	47183.8	2.62135	newly 50 weeks cumul	0.1	60.6757	{27., 107.}	60575.7	2.72831
9	newly 3 weeks spec	22.	14.3651	{6., 25.}	51.7316	0.326783	newly 3 weeks cumul	56.	55.9206	{24., 108.}	47.2506	0.215967	newly 5 weeks spec	13.	10.8889	{4., 23.}	56.7766	0.303415	newly 5 weeks cumul	88.	80.1587	{32., 146.}	48.1241	0.232487	newly 46 weeks cumul	139.	138.825	{48., 248.}	58.3647	0.253818
10	newly 3 weeks spec	10.	14.6949	{3., 31.}	84.5763	0.266573	newly 3 weeks cumul	37.	53.2881	{18., 100.}	77.5538	0.247485	newly 5 weeks spec	2.	10.4915	{2., 22.}	434.746	0.585983	newly 5 weeks cumul	45.	74.8136	{24., 143.}	92.919	0.254382	newly 46 weeks cumul	50.	111.678	{32., 209.}	140.712	0.3028
11	newly 3 weeks spec	17.	16.3061	{6., 34.}	43.9376	0.222954	newly 3 weeks cumul	63.	55.3265	{17., 104.}	41.7234	0.230071	newly 5 weeks spec	10.	10.0408	{3., 20.}	59.1837	0.291348	newly 5 weeks cumul	90.	77.7551	{25., 151.}	42.8118	0.235011	newly 43 weeks cumul	187.	126.918	{40., 246.}	44.8325	0.298391
12	newly 3 weeks spec	0.1	5.07463	{1., 10.}	4980.6	1.55468	newly 3 weeks cumul	10.	21.1045	{9., 33.}	117.313	0.293671	newly 5 weeks spec	0.1	2.46269	{0., 5.}	2407.46	1.08558	newly 5 weeks cumul	10.	26.7612	{11., 43.}	169.403	0.368711	newly 51 weeks cumul	10.	31.4627	{13., 47.}	216.418	0.437061
13	newly 3 weeks spec	9.	15.3333	{4., 35.}	98.8304	0.285769	newly 3 weeks cumul	45.	55.3509	{21., 113.}	56.9201	0.206169	newly 5 weeks spec	9.	9.4386	{3., 22.}	66.4717	0.316595	newly 5 weeks cumul	69.	77.0526	{28., 152.}	54.3351	0.212435	newly 46 weeks cumul	95.	118.421	{36., 258.}	63.6565	0.222275
14	newly 3 weeks spec	8.	14.3896	{6., 24.}	92.5325	0.253894	newly 3 weeks cumul	35.	57.9351	{32., 94.}	72.2078	0.202022	newly 5 weeks spec	7.	8.35065	{3., 14.}	53.8033	0.215984	newly 5 weeks cumul	50.	76.7662	{41., 117.}	64.1299	0.18798	newly 51 weeks cumul	59.	102.091	{54., 154.}	79.3749	0.214371
15	newly 3 weeks spec	1.	6.06849	{1., 12.}	517.808	0.679819	newly 3 weeks cumul	11.	24.4247	{6., 53.}	140.224	0.339962	newly 5 weeks spec	1.	4.27397	{0., 9.}	352.055	0.620393	newly 5 weeks cumul	12.	34.3014	{10., 75.}	197.489	0.404736	newly 45 weeks cumul	13.	49.5479	{15., 110.}	288.514	0.483016
16	newly 3 weeks spec	17.	11.6667	{4., 18.}	46.2418	0.288312	newly 3 weeks cumul	40.	48.0278	{23., 73.}	46.5278	0.172612	newly 5 weeks spec	8.	7.02778	{2., 13.}	54.8611	0.313302	newly 5 weeks cumul	60.	63.4028	{29., 93.}	42.338	0.178622	newly 51 weeks cumul	89.	82.25	{38., 114.}	41.2921	0.199314
17	newly 3 weeks spec	3.	7.25676	{2., 15.}	156.306	0.368349	newly 3 weeks cumul	13.	32.2568	{14., 54.}	152.287	0.337825	newly 5 weeks spec	2.	4.39189	{1., 9.}	137.162	0.361919	newly 5 weeks cumul	18.	42.6486	{18., 84.}	141.742	0.323853	newly 51 weeks cumul	18.	52.3649	{20., 106.}	193.318	0.398928
18	newly 3 weeks spec	24.	16.2075	{5., 28.}	52.4371	0.356196	newly 3 weeks cumul	73.	65.4906	{25., 126.}	47.7643	0.251548	newly 5 weeks spec	26.	9.49057	{2., 20.}	66.4006	0.606332	newly 5 weeks cumul	128.	87.717	{34., 181.}	50.5749	0.317707	newly 51 weeks cumul	297.	113.981	{48., 230.}	64.0112	0.519094
19	newly 3 weeks spec	13.	9.30909	{3., 22.}	61.3986	0.39811	newly 3 weeks cumul	52.	36.7091	{13., 72.}	56.8881	0.339737	newly 5 weeks spec	8.	5.81818	{1., 14.}	65.9091	0.446306	newly 5 weeks cumul	69.	50.0727	{16., 101.}	57.365	0.334822	newly 44 weeks cumul	105.	76.9636	{23., 144.}	61.0216	0.357807
20	newly 3 weeks spec	5.	10.9661	{3., 20.}	134.237	0.319471	newly 3 weeks cumul	13.	41.661	{15., 82.}	224.12	0.428574	newly 5 weeks spec	1.	6.98305	{1., 14.}	601.695	0.699775	newly 5 weeks cumul	19.	57.1017	{20., 108.}	205.531	0.404386	newly 44 weeks cumul	36.	86.7627	{27., 162.}	153.531	0.332227
21	newly 3 weeks spec	15.	9.30645	{3., 16.}	56.2366	0.384085	newly 3 weeks cumul	44.	37.5806	{16., 65.}	48.4604	0.249667	newly 5 weeks spec	7.	4.8871	{1., 9.}	56.9124	0.44717	newly 5 weeks cumul	62.	49.1613	{18., 83.}	49.3236	0.269921	newly 51 weeks cumul	100.	63.4839	{25., 107.}	51.3548	0.329559
22	newly 3 weeks spec	1.	11.0667	{3., 22.}	1010.	0.880084	newly 3 weeks cumul	13.	42.8167	{12., 83.}	235.513	0.415191	newly 5 weeks spec	0.1	7.75	{1., 14.}	7660.	1.65437	newly 5 weeks cumul	13.	61.1	{16., 110.}	372.564	0.542645	newly 45 weeks cumul	13.	90.	{22., 157.}	593.59	0.680478
23	newly 3 weeks spec	12.	14.3043	{4., 29.}	64.372	0.260873	newly 3 weeks cumul	38.	51.0145	{19., 103.}	64.6834	0.216851	newly 5 weeks spec	6.	8.4058	{2., 18.}	80.6763	0.330841	newly 5 weeks cumul	50.	70.2609	{25., 143.}	70.2029	0.227315	newly 47 weeks cumul	82.	103.29	{38., 210.}	63.1849	0.222598
24	newly 3 weeks spec	7.	6.55172	{1., 15.}	80.2956	0.469132	newly 3 weeks cumul	21.	25.6207	{6., 51.}	76.3547	0.291735	newly 5 weeks spec	2.	4.82759	{0., 9.}	184.483	0.467635	newly 5 weeks cumul	24.	36.0172	{9., 69.}	90.4454	0.2705	newly 44 weeks cumul	30.	52.4828	{12., 99.}	110.	0.285968
25	newly 3 weeks spec	3.	10.	{3., 21.}	241.935	0.43848	newly 3 weeks cumul	19.	36.871	{12., 72.}	112.394	0.276465	newly 5 weeks spec	2.	7.27419	{1., 13.}	281.452	0.479812	newly 5 weeks cumul	27.	53.1774	{16., 99.}	115.711	0.277691	newly 42 weeks cumul	28.	92.5484	{24., 145.}	238.364	0.39176
26	newly 3 weeks spec	6.	6.41176	{1., 13.}	61.2745	0.336261	newly 3 weeks cumul	26.	26.5147	{10., 54.}	50.7353	0.242312	newly 5 weeks spec	13.	3.73529	{0., 9.}	71.7195	0.777461	newly 5 weeks cumul	53.	35.1618	{14., 77.}	49.1398	0.318243	newly 49 weeks cumul	100.	45.8529	{19., 98.}	57.	0.43886
27	newly 3 weeks spec	0.1	10.6618	{2., 22.}	10564.7	1.85086	newly 3 weeks cumul	0.1	41.9559	{14., 78.}	41855.9	2.5137	newly 5 weeks spec	0.1	6.48529	{1., 12.}	6397.06	1.60754	newly 5 weeks cumul	0.1	56.6471	{18., 104.}	56547.1	2.63998	newly 48 weeks cumul	0.1	75.2794	{23., 135.}	75179.4	2.7614
28	newly 3 weeks spec	18.	15.6866	{8., 24.}	42.2056	0.212126	newly 3 weeks cumul	61.	60.7015	{29., 91.}	40.4208	0.176865	newly 5 weeks spec	6.	9.40299	{2., 17.}	85.0746	0.311076	newly 5 weeks cumul	78.	82.7612	{37., 132.}	42.9583	0.178206	newly 51 weeks cumul	104.	110.716	{48., 167.}	45.4363	0.188417
29	newly 3 weeks spec	6.	6.24138	{1., 14.}	71.2644	0.349087	newly 3 weeks cumul	22.	24.	{7., 55.}	71.7868	0.301313	newly 5 weeks spec	6.	4.75862	{0., 12.}	73.5632	0.538862	newly 5 weeks cumul	37.	34.4483	{10., 86.}	69.2451	0.334513	newly 44 weeks cumul	89.	52.5172	{11., 129.}	70.1279	0.488935
30	newly 3 weeks spec	5.	10.3898	{2., 22.}	143.729	0.371688	newly 3 weeks cumul	19.	35.7288	{10., 75.}	115.165	0.283647	newly 5 weeks spec	1.	6.0339	{0., 13.}	527.119	0.653062	newly 5 weeks cumul	23.	49.4237	{13., 99.}	136.404	0.298195	newly 45 weeks cumul	36.	72.1525	{19., 134.}	129.614	0.300557
31	newly 3 weeks spec	5.	8.32258	{2., 14.}	92.9032	0.270948	newly 3 weeks cumul	36.	32.0484	{12., 54.}	48.4319	0.243822	newly 5 weeks spec	7.	6.09677	{1., 11.}	63.1336	0.410824	newly 5 weeks cumul	48.	45.2903	{17., 72.}	49.1263	0.231251	newly 46 weeks cumul	144.	68.6774	{20., 109.}	66.2186	0.483452
32	newly 3 weeks spec	16.	25.6885	{12., 45.}	72.8484	0.220859	newly 3 weeks cumul	65.	92.9508	{41., 144.}	57.3266	0.193397	newly 5 weeks spec	15.	19.5246	{8., 33.}	58.3607	0.205551	newly 5 weeks cumul	94.	135.984	{63., 228.}	59.5221	0.197078	newly 48 weeks cumul	156.	220.426	{94., 383.}	61.7486	0.201684
33	newly 3 weeks spec	6.	13.5915	{5., 26.}	135.446	0.314002	newly 3 weeks cumul	27.	55.3662	{28., 86.}	107.46	0.264874	newly 5 weeks spec	2.	7.59155	{2., 14.}	288.028	0.517323	newly 5 weeks cumul	33.	73.9577	{34., 116.}	126.504	0.298923	newly 51 weeks cumul	47.	96.7606	{44., 156.}	110.668	0.267282
34	newly 3 weeks spec	10.	7.42857	{1., 18.}	72.0635	0.493268	newly 3 weeks cumul	29.	27.7778	{8., 59.}	65.8456	0.312839	newly 5 weeks spec	9.	5.63492	{1., 9.}	73.0159	0.582761	newly 5 weeks cumul	48.	39.4921	{11., 81.}	67.5265	0.36312	newly 43 weeks cumul	102.	60.873	{16., 118.}	68.8609	0.462361
35	newly 3 weeks spec	0.1	11.6986	{4., 21.}	11598.6	1.99442	newly 3 weeks cumul	3.	47.7945	{22., 81.}	1493.15	1.15146	newly 5 weeks spec	0.1	6.56164	{1., 11.}	6467.12	1.67696	newly 5 weeks cumul	3.	62.8219	{30., 105.}	1994.06	1.26725	newly 51 weeks cumul	3.	80.9726	{37., 149.}	2599.09	1.3753
36	newly 3 weeks spec	0.1	1.56944	{0., 4.}	1538.89	0.840928	newly 3 weeks cumul	0.1	7.40278	{2., 15.}	7308.33	1.70133	newly 5 weeks spec	0.1	0.694444	{0., 2.}	708.333	0.497062	newly 5 weeks cumul	0.1	8.79167	{2., 18.}	8694.44	1.78232	newly 52 weeks cumul	0.1	9.97222	{2., 20.}	9875.	1.82678
37	newly 3 weeks spec	18.	7.45	{1., 15.}	67.3148	0.622236	newly 3 weeks cumul	45.	26.8833	{8., 46.}	60.3333	0.399966	newly 5 weeks spec	10.	5.1	{0., 10.}	69.6667	0.664503	newly 5 weeks cumul	67.	38.1667	{11., 69.}	61.194	0.42551	newly 42 weeks cumul	100.	70.1167	{18., 121.}	72.05	0.427324
38	newly 3 weeks spec	3.	5.93939	{2., 11.}	114.141	0.304658	newly 3 weeks cumul	8.	26.3333	{12., 49.}	230.303	0.459259	newly 5 weeks spec	0.1	3.33333	{0., 7.}	3260.61	1.30449	newly 5 weeks cumul	8.	33.7576	{16., 61.}	322.348	0.566955	newly 50 weeks cumul	8.	42.3333	{19., 76.}	429.545	0.66079
39	newly 3 weeks spec	6.	14.1493	{5., 25.}	142.786	0.325669	newly 3 weeks cumul	26.	52.0299	{22., 78.}	105.855	0.269594	newly 5 weeks spec	6.	8.68657	{3., 17.}	75.6219	0.266488	newly 5 weeks cumul	37.	72.0149	{32., 117.}	100.121	0.255087	newly 46 weeks cumul	74.	106.493	{45., 185.}	63.7152	0.19652
40	newly 3 weeks spec	9.	4.11111	{0., 9.}	64.1975	0.615314	newly 3 weeks cumul	26.	18.1528	{5., 33.}	51.3355	0.326177	newly 5 weeks spec	4.	1.81944	{0., 4.}	66.3194	0.790653	newly 5 weeks cumul	37.	22.5139	{6., 44.}	54.1667	0.378207	newly 52 weeks cumul	38.	26.0417	{7., 51.}	54.788	0.365546
41	newly 3 weeks spec	0.1	9.41333	{2., 18.}	9313.33	1.85179	newly 3 weeks cumul	9.	35.9733	{13., 65.}	299.704	0.520401	newly 5 weeks spec	0.1	4.85333	{1., 10.}	4756.	1.55921	newly 5 weeks cumul	9.	47.6667	{17., 83.}	429.63	0.642193	newly 49 weeks cumul	9.	62.84	{22., 115.}	598.222	0.757864
42	newly 3 weeks spec	13.	12.0725	{4., 22.}	51.0591	0.260018	newly 3 weeks cumul	42.	46.8116	{19., 84.}	49.3444	0.195313	newly 5 weeks spec	12.	7.62319	{2., 15.}	55.314	0.379954	newly 5 weeks cumul	62.	64.2464	{28., 119.}	47.6625	0.200811	newly 49 weeks cumul	154.	87.4928	{33., 159.}	55.1007	0.363904
43	newly 3 weeks spec	2.	9.26984	{2., 17.}	371.429	0.573273	newly 3 weeks cumul	22.	35.7143	{12., 61.}	85.7143	0.247171	newly 5 weeks spec	4.	6.96825	{2., 13.}	105.952	0.306022	newly 5 weeks cumul	31.	49.8095	{15., 85.}	83.7174	0.240485	newly 42 weeks cumul	85.	86.6667	{23., 159.}	66.9468	0.286632
44	newly 3 weeks spec	4.	11.6842	{3., 22.}	203.289	0.40921	newly 3 weeks cumul	11.	44.0789	{17., 82.}	301.196	0.513946	newly 5 weeks spec	3.	6.98684	{2., 13.}	146.93	0.335289	newly 5 weeks cumul	24.	59.9211	{22., 106.}	154.057	0.328159	newly 50 weeks cumul	29.	81.3289	{32., 135.}	183.893	0.363864
45	newly 3 weeks spec	11.	10.1538	{4., 18.}	51.6084	0.25522	newly 3 weeks cumul	31.	41.4154	{18., 67.}	57.0223	0.185958	newly 5 weeks spec	14.	6.84615	{2., 11.}	60.1099	0.507701	newly 5 weeks cumul	50.	57.1846	{23., 93.}	49.2	0.186775	newly 47 weeks cumul	85.	79.8462	{32., 127.}	46.5339	0.218562
46	newly 3 weeks spec	18.	13.9194	{5., 28.}	47.043	0.279163	newly 3 weeks cumul	58.	49.5161	{20., 83.}	43.7152	0.235304	newly 5 weeks spec	11.	9.37097	{3., 18.}	50.2933	0.307279	newly 5 weeks cumul	91.	70.5484	{28., 120.}	44.0269	0.254553	newly 46 weeks cumul	196.	118.5	{37., 208.}	54.5178	0.359556
47	newly 3 weeks spec	0.1	4.73333	{1., 11.}	4650.	1.4451	newly 3 weeks cumul	0.1	22.2833	{5., 39.}	22183.3	2.20317	newly 5 weeks spec	0.1	2.61667	{0., 5.}	2563.33	1.08073	newly 5 weeks cumul	0.1	28.1833	{7., 53.}	28083.3	2.29881	newly 52 weeks cumul	0.1	32.85	{7., 64.}	32750.	2.36749
48	newly 3 weeks spec	5.	5.875	{2., 10.}	58.125	0.261371	newly 3 weeks cumul	29.	23.8438	{9., 45.}	44.181	0.241066	newly 5 weeks spec	5.	3.42188	{0., 7.}	55.3125	0.455693	newly 5 weeks cumul	37.	32.1563	{14., 60.}	43.4966	0.230754	newly 46 weeks cumul	57.	43.4688	{16., 78.}	43.5307	0.259098
49	newly 3 weeks spec	13.	8.0274	{2., 14.}	62.6976	0.430071	newly 3 weeks cumul	31.	31.8356	{10., 51.}	55.5457	0.234354	newly 5 weeks spec	3.	4.20548	{0., 7.}	94.0639	0.430289	newly 5 weeks cumul	42.	42.1644	{15., 63.}	54.5336	0.230496	newly 50 weeks cumul	46.	54.6986	{17., 82.}	59.9464	0.210323
50	newly 3 weeks spec	11.	16.4627	{7., 29.}	69.7422	0.20586	newly 3 weeks cumul	42.	59.8955	{28., 109.}	58.6709	0.183009	newly 5 weeks spec	10.	10.9104	{3., 23.}	60.4478	0.277484	newly 5 weeks cumul	58.	83.1493	{35., 144.}	60.808	0.18809	newly 48 weeks cumul	98.	122.612	{47., 232.}	55.0868	0.196589
